# Infection prevention and control in 2030: a first qualitative survey by the Crystal Ball Initiative

**DOI:** 10.1186/s13756-024-01431-3

**Published:** 2024-08-13

**Authors:** Hugo Sax, Jonas Marschall, and the Crystal Ball Initiative, Sue Barnes, John M. Boyce, Suzanne F. Bradley, Dale Fisher, Andrea Grisold, Stephan Harbarth, Anita Huis, Elaine Larson, Andie Lee, Grace Lee, Yves Longtin, Jean-Christophe Lucet, Eli Perencevich, Simone Scheithauer, Julie Storr, Jason-Anthony Tetro, Erich Tschirky, Diana Vilar-Compte

**Affiliations:** 1 Department of Infectious Diseases, Bern University Hospital and University of Bern, Friedbuehlstrasse 53, CH-3010 Bern, Switzerland; 2 Division of Infectious Diseases, John T Milliken Department of Internal Medicine, Washington University School of Medicine, 4523 Clayton Ave, St. Louis, MO 63110 USA

**Keywords:** Infection prevention, Infection control (MeSH term), Futures studies, Futurists, Qualitative research, Trends, Survey, Expert opinion

## Abstract

**Background:**

Healthcare delivery is undergoing radical changes that influence effective infection prevention and control (IPC). Futures research (short: Futures), the science of deliberating on multiple potential future states, is increasingly employed in many core societal fields. Futures might also be helpful in IPC to facilitate current education and organisational decisions. Hence, we conducted an initial survey as part of the IPC Crystal Ball Initiative.

**Methods:**

In 2019, international IPC experts were invited to answer a 10-item online questionnaire, including demographics, housekeeping, and open-ended core questions (Q) on the “status of IPC in 2030” (Q1), “people in charge of IPC” (Q2), “necessary skills in IPC” (Q3), and “burning research questions” (Q4). The four core questions were submitted to a three-step inductive and deductive qualitative content analysis. A subsequent cross-case matrix produced overarching leitmotifs. Q1 statements were additionally coded for sentiment analysis (positive, neutral, or negative).

**Results:**

Overall, 18 of 44 (41%) invited experts responded (from 11 countries; 12 physicians, four nurses, one manager, one microbiologist; all of them in senior positions). The emerging leitmotifs were “System integration”, “Beyond the hospital”, “Behaviour change and implementation”, “Automation and digitalisation”, and “Anticipated scientific progress and innovation”. The statements reflected an optimistic outlook in 66% of all codes of Q1.

**Conclusions:**

The first exercise of the IPC Crystal Ball Initiative reflected an optimistic outlook on IPC in 2030, and participants envisioned leveraging technological and medical progress to increase IPC effectiveness, freeing IPC personnel from administrative tasks to be more present at the point of care and increasing IPC integration and expansion through the application of a broad range of skills. Enhancing participant immersion in future Crystal Ball Initiative exercises through simulation would likely further increase the authenticity and comprehensiveness of the envisioned futures.

## Introduction

Healthcare delivery faces significant challenges and disruptive changes [1]. These challenges have triggered an interest in possible future developments and strategic discussions on a global level. For example, the Future Today Institute (https://futuretodayinstitute.com) considers public health as one of the macro forces that provide important signals for predicting technological trends. One of the United Nations’ Sustainable Development Goals is to ensure healthy lives and promote well-being for all by 2030 (https://sdgs.un.org/goals). The experience of the COVID-19 pandemic has accelerated this process and propelled IPC to centre stage. However, healthcare-associated infections (HAI) continue to cause patient suffering and loss of life, prolong medical treatment, and accelerate antimicrobial resistance, despite their preventability [2]. Over the last 25 years, numerous large-scale initiatives have been launched to address these problems, but they tend to be reactive and not focused on the future.

Looking into possible, more distant futures and spelling out visions for IPC might help guide current efforts and open the mind to see the present-day challenges and positively influence developments and uptake of innovation. This could benefit decision-making for designing workforce training, revising healthcare organisational structures, investing in architectural and electronic infrastructure, and advancing science. Futures research, or Futures, has evolved over the last 20 years into a field of scientific inquiry into what the world might look like far ahead [3]. As the most productive methodologic innovation, Futures has moved from “predicting the future to mapping alternative futures […], both at external collective levels and individual inner levels” [4]. The field has further benefited from growing operational experience and further innovations, e.g., the real-time Delphi method by The Millennium Project [5] or the Experiential Futures ‘Show and tell’ approach to “…design situations that help us understand possible futures by visiting them” [6]. Not surprisingly, healthcare holds a privileged place in Futures research [1].

The interest in a more radical and systematic exploration of the potential futures of IPC was initially triggered by strategic sessions within Swissnoso, the Swiss Centre for Infection Prevention (https://www.swissnoso.ch), and evolved from there in informal discussions with experts at international conferences and other occasions. Due to the great interest, we created the Crystal Ball Initiative as an ongoing platform for Futures research in IPC open to interested parties and further research initiatives [7]. Here, we present the qualitative analysis of the first survey conducted in 2019, shortly before the onset of the SARS-CoV-2 pandemic.

## Methods

### Study design

A transversal qualitative survey study based on open questions [8].

### Participants

In January 2019, we invited a purposive sample of 44 multinational Infection Prevention and Control (IPC) experts with nursing and physician backgrounds to participate. They were selected based on their innovative approaches to IPC as manifested by their leadership in scientific publications, global and national leadership positions, contributions to international and national organisations and initiatives, professional conferences as speakers, and social media presence.

### Data collection

The online questionnaire was created to anticipate future developments in IPC in research and practice relevant to strategic development for IPC experts, funding agencies, policymakers and healthcare managers. Brevity and clarity were design requirements to increase the return rate with busy experts. Various formulations were developed iteratively by a working group of IPC expert nurses, physicians, a psychologist, and a data analyst. IPC volunteer experts were asked to test the questionnaire versions using a ‘think-aloud’ technique, i.e., constantly voicing their thoughts while reading and answering questions. This process led to four free-text core questions (Q1-Q4) and seven “housekeeping” questions (Table 1). The online self-administered survey (Survey Monkey by momentive.ai) was opened in January 2019 with an invitation sent to the 44 selected IPC experts – followed by a reminder in February 2019 – and closed in March 2019.

**Table 1:** Survey questions

Core questions	
Q1	What will infection prevention in healthcare settings look like in 2030 (thinking of a typical day in healthcare)?
Q2	In whose hands will infection prevention in healthcare settings lay in 2030? Who will be responsible?
Q3	What skills and knowledge will be necessary to execute infection prevention in healthcare settings successfully in 2030?
Q4	What are the most burning (research) questions regarding infection prevention in healthcare settings that should/will be answered by 2030?
Supportive questions	
Q5	What publications or other knowledge resources could you suggest that contain relevant information to the future of IPC?
Q6	Would you like to suggest individuals we should ask to participate as co-authors in this study? Please indicate your name and, ideally, email.
Q7	Your job title(s)
Q8	Your affiliation(s)
Q9	Would you be available for a 20–30-min interview to deepen the topic?
Q10	Would you accept to be a co-author of a publication of the results of this inquiry according to the ICMJE requirements for authorship?
Q11	Do you have suggestions on how to enhance this inquiry methodologically?

### Analysis

**Fig. 1:**
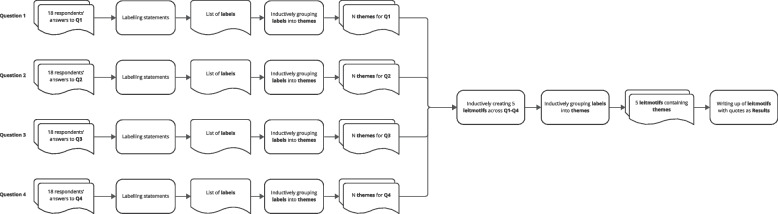
The inductive and deductive qualitative analysis procedure

Two researchers performed a structured qualitative analysis (HS, JM) of the core questions. First, one author (HS) labelled each statement in the respondents’ answers to core questions Q1–Q4 separately with a *code*, favouring the respondent’s terminology for code names (Fig. 1), while a second author (JM) ‘proofread’ the coding, and concerns were resolved in a bilateral discussion. These codes were then inductively grouped into *themes* within each core question. Next, themes were further grouped under “leitmotifs” that held true across the core questions. The two authors (HS, JM) additionally evaluated all coded statements in the answers to Q1, whether they were formulated in a positive, negative, or neutral tone for sentiment analysis. Concerns were resolved through discussion. Finally, the results were submitted to the respondents for ‘member checking’ in the form of an early manuscript draft of this publication to ensure that the results were complete and accurate [8].

## Results

**Fig. 2:**
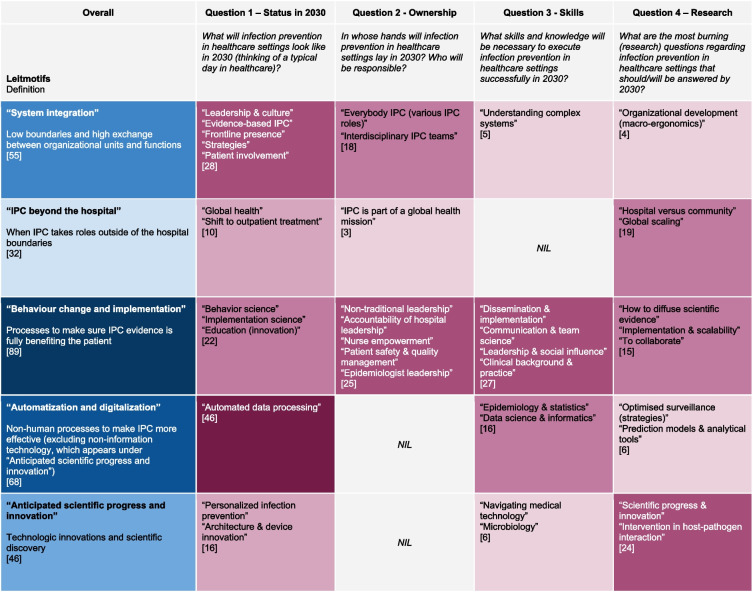
Heat matrix of core questions, themes, and leitmotifs. Legend: The number of quotes is indicated in square brackets in each cell of the heat matrix matched by colour intensity; IPC, infection prevention and control

Overall, 18 of the 44 (41%) invited responded; six from the USA, two from Canada and Switzerland, and one each from Australia, Austria, France, Germany, Mexico, Singapore, The Netherlands, and The United Kingdom; 12 were physicians, four were nurses, one had a healthcare management background, and one was a microbiologist by education, all with higher education and in senior positions. The cross-question analysis produced the five leitmotifs (Fig. 2) subsequently presented in detail. The subjective connotation of the answers to Q1 in terms of the desirability of future states of IPC, was positive in 66%, negative in 15%, and neutral in 19% of cases. Based on Q4, Table 2 highlights suggested skills and roles for IPC in 2030 and Table 3 lists essential research for the envisioned futures.

**Table 2:** Suggested skills and roles for IPC in 2030 (survey question 3)

Skills, know-how	Role, profession, education
Infection prevention	Infectious prevention practitioner, nurse, physician
Infectious diseases	Infectious disease physician
Epidemiology	Epidemiologist
Public health	Master in public health (MPH)
Engineering, human factor engineering	Human factors engineer
Information technology	Information Technology specialist, programmer, hardware specialist
Data science with modelling skills	Data scientist
Project management	Project manager
Communication	Communication expert
Social media	Communication expert
Education	Educator
Change management	Change manager, quality improvement specialist
Performance improvement	Change manager, quality improvement specialist
Implementation science	Implementation scientist
Behaviour-change techniques	Psychologist
Understanding complex systems	Systems engineer, complexity scientist

**Table 3:** Research question to be answered for IPC by 2030 (survey question 4)

Research themes	Research topics
Organisational development	How can failure-free systems and devices be designed and promoted?
	What is the optimal IPC program for single- and multi-hospital settings?
	What is the potential role of IPC leadership in healthcare beyond IPC?
Hospital—community interface	What is the role of IPC in primary care and outpatient settings?
	How do MDROs impact healthcare in LMICs and communities with political, sanitation, and food safety challenges?
	How does absent or substandard IPC affect people's trust in healthcare institutions?
	What is the impact of "world changes" (climate, migration, natural disasters, water, emerging technologies, emerging diseases) on IPC?
Global scaling, including for MDRO	How prepared are we to manage public health emergencies?
	What are the best methods to train, evaluate, and certify IPC experts?
	How do healthcare delivery changes impact healthcare-associated infections?
	What role can and should IPC play in the prevention and management of non-communicable diseases?
	What role does IPC play in the United Nations' 17 Sustainable Development Goals?
Economics and overall health outcome	How cost-effective is IPC in the context of increasing healthcare costs?
	What is the importance and role of IPC in overall patient outcomes?
Diffusion of IPC evidence	How can the information on vaccination benefits be effectively communicated?
	What knowledge infrastructure is needed for IPC diffusion?
	How can IPC knowledge be best disseminated across the healthcare setting?
Implementation and scalability	What are the most effective IPC behaviour change strategies?
	How can successful IPC strategies be scaled up effectively?
	What are the best methods and frameworks for implementation science?
	How can successful IPC efforts be sustained?
Collaboration	What is the intersection between antimicrobial stewardship and IPC?
	How can collaboration be established across IPC think tanks, innovation hubs, and initiatives?

Legend: The Research themes in this table refer to the themes established within the core question Q4 during the qualitative analysis (s. Figure 2). *IPC* infection prevention and control, *HAI* healthcare-associated infection, *MDRO* multi-drug-resistant organisms, *LMIC* low to middle income countries

### “System integration”

These days, IPC generally constitutes a separate unit of IPC professionals in hospitals. However, participants questioned this traditional thinking and projected an increase in organisational system integration. This extends to economics and quality of care measures. IPC has also to adapt to the accelerating shift of medical care to outpatient and long-term settings.

Quote: “Infection prevention must be perceived as a system rather than a specific programme.”

By 2030, IPC leaders will play an integrative role in patient safety by connecting the current players. One participant foresaw IPC leaders proactively breaking traditional silos and occupying influential roles as advocates for safer healthcare. However, in a pessimistic outlook, IPC teams will be absorbed by antibiotic stewardship or quality management programs, which could be associated with an undesirable loss of IPC-specific know-how.

Quote: “IPC nirvana in 2030 will see IPC integrated across every aspect of healthcare and the role of IPC experts shrinking to be more influencers and leaders and fewer coveters of technical know-how. IPC is and will be stronger and more influential if we (the IPC community) secure a strong voice at the global health table. If we do not, others jump in and take our place, and almost without exception, those “others” lack experience/expertise, capability, and competence to talk with depth and understanding on IPC.”

IPC teams need a broader range of knowledge and skills than today (Table 2). Among these, understanding complex systems was stated explicitly.

Quote: “I think the infection prevention and control/hospital epidemiology field will need to rethink the skillsets needed on the team to optimise the success of programs.”

Quote: “Knowledge about complex care practices.”

A strong emphasis was put on the presence of nurses and physicians in IPC teams and their presence as observers and coaches, face-to-face with care providers. In addition, the future patient will have a role in IPC, will be educated and empowered as a healthcare ‘consumer’ and will be involved in co-creating the care processes and IPC measures.

Research and development will provide the best organisational models and integration of IPC in various healthcare contexts, such as the increasingly dominant outpatient care sector (Table 3). Furthermore, since financial constraints will have become even more relevant for healthcare institutions by 2030, IPC constitutes a driving force in cost-saving based on a more robust calculation of the costs imposed by HAI and costs generated by prevention measures than is the case today.

Quote: “2030 is just 11 years away. Looking back 11 years ago to 2008 as a comparator, I think we have to be realistic and conclude that IPC in 2030 will be fairly similar to what it is today.”

System integration also concerns a better understanding of HAI and the ever-expanding role of the microbiome. Microbiology’s role will be considered more holistically, beyond its current focus on single pathogens, accounting for, e.g., the transmission of virulence factors and resistance elements between patients, and between patient and environmental microbiota.

### “Beyond the hospital”

Some respondents foresaw that by 2030 IPC responsibilities and activities will have transcended today’s institutional boundaries.

Quote: “IPC has to move out of its comfort zone and get around the table with health systems and health security leaders – we need a serious voice – not others talking on our behalf. In every country of the world, we need IPC leaders who could fulfil the roles of chief medical officer or chief nursing officer within ministries of health.”

In an undesirable future, however, the heightened governmental interest in IPC could become dominant and lead to over-regulation and poor adaptation of policies according to local needs.

Per the responders, challenges to be tackled by IPC in 2030 include climate change, human migration, emerging diseases, political influence, global health threats, lack of potable water, emerging technologies, and the slow uptake of modern IPC in low-to-middle-income countries.

Quote: “How are we prepared to deal with public health and national or international emergencies?”

Quote: “IPC does not yet “own” the low- and middle-income countries space.”

However, some respondents suggested that IPC in low- and middle-income will be better understood in 2030 thanks to dedicated research.

### “Behaviour change and implementation”

By 2030, bench-to-bedside translation will be a central activity of IPC teams. IPC will produce globally recognised behavioural and implementation scientists. They will use behaviour change instruments such as ‘nudging’ and Plan-Do-Study-Act (PDSA) cycles for continuous system improvement more readily. Additionally, performance monitoring and feedback will be optimised, and research will have identified the most effective implementation frameworks.

Dissemination of best IPC practices will benefit from innovative educational techniques such as simulation [9], e-learning, virtual reality [10], and ‘serious games’ [11]. Interventions will be more successful because they can be systematically based on better scientific evidence that will have accumulated. IPC content will be an established part of undergraduate curricula.

Quote: “[…] What is much harder is figuring out “how” we will manage to implement these goals. So “soft skills” will be extremely important. Human relationships, diplomacy, compromise, listening, team building capacity, leadership, showing gratefulness, etc.”

Leadership enabled with strong social and communication skills will be essential for the sustainable success of IPC within healthcare systems.

Quote: “First and foremost, leadership development and an understanding of the value of developing strong and effective IPC leaders, empowered to take IPC where it needs to go and to get IPC understood by those who need to understand it.”

As mentioned in the leitmotif “System integration”, patient education and participation in IPC will support the implementation of preventive measures in two ways, by their own adherence, e.g., by toothbrushing for non-ventilator-associated pneumonia prevention, and by reminding healthcare providers in case of forgetting to apply their preventive tasks.

### “Automation and digitalisation”

Data will be accessible to IPC teams through automated analytics and queries. Typical data sources include electronic medical records, laboratories, patient feedback, and multiple sensors. In addition, invasive devices are electronically identified and contribute to risk management on individual and institutional levels.

Quote: “The surveillance for infections will be fully automated, leaving the IP team free to put eyes on high-risk areas to identify risks, assess compliance with protocols, and provide just-in-time coaching. Video auditing will assist with the eyes on the component of the job - to enhance the reach of the human IPC resources. Radio Frequency Identification (RFID) or similar tech will be added to all indwelling devices and interact with the electronic medical record to automate device day counts, which will feed into the automated surveillance system. IPC protocols will be embedded into electronic medical records to provide real-time clinical guidance. Decision support regarding testing and treatment for infections will also be a component of the medical record.”

Intelligent surveillance systems will continuously scan data to highlight abnormal phenomena, e.g., an increase in the incidence of bloodstream infections, and point to system failures at an early stage. Caregivers will receive recommendations for personalised preventive measures within their patients’ electronic medical records. IPC teams will base their work on dashboards with highly automated data pre-processing and respond to real-time alerts. For example, dashboards will highlight daily ‘hot spots’ and trigger immediate measures. Context- and time-sensitive mobile educational IPC content will reach caregivers on the go. Digital coaching systems will observe caregivers during their daily work and provide real-time feedback on adherence issues.

This shift to automation calls for new IPC skill sets and work routines. Here, the views of survey participants diverged. In one possible future, artificial intelligence will make human epidemiologic know-how obsolete, shifting the focus to socio-adaptive expertise and allowing teams to spend their time in hospital wards for coaching and problem scouting. In an alternative future, however, IPC teams will require much more epidemiology and data science skills than today.

Quote: “Familiarity and expertise in data management, statistical analysis of data, epidemiological principles, use of artificial intelligence, methods for recognition and investigation of clusters or outbreaks of HAIs, knowledge of antimicrobial resistance patterns of pathogens.”

Artificial intelligence will also support bench-to-bedside translation. IPC-relevant scientific evidence will be captured and funnelled by machine learning to IPC teams and even frontline care providers in an actionable format, constantly keeping policies up-to-date.

Research involving automated surveillance of microbiology results will intensify and lead to a better understanding of the importance of patient, caregiver, and environmental microbiota and transmission pathways. Advances in calculation power, machine learning, and rapid genetic typing will enable real-time mapping of colonisation and infections. HAI definitions will have to adapt to this automation. Point-of-care testing will replace laboratory-based testing and will be guiding patient isolation.

More detailed digital information on each patient reduces the need for physical proximity with patients, which reduces pathogen transmission.

### “Anticipated scientific progress and innovation”

Dedicated research will lead to a better understanding of transmission pathways, which will inform hospital and policy design.

Quote:** “**What is the relative importance of the various routes of transmissions (direct contact vs indirect contact-environment vs. droplet vs airborne)?”

Quote: “What is the relative value of various infection control strategies in the prevention of infections and the prevention of the spread of multi-resistant organisms (e.g., basic precautions vs additional precautions vs antibiotic stewardship)?”

Scientific evidence will transform hospital design, e.g., single rooms will be the standard, redesigned shower drains and sewage systems will prevent pathogen aerosolisation, and improved design of personal protective equipment will guarantee caregiver safety.

IPC teams will have adopted health technology more profoundly. An improved concept for hand hygiene will likely have emerged, facilitating caregiver training and behaviour. Techniques to render pathogens visible will support adherence to IPC measures further. Hand hygiene products could be enhanced with microbial remnant-interacting components, given their benefit for HAI prevention will have been proven.

In one imagined future, vaccines against hospital pathogens will have revolutionised IPC based on a better understood host–pathogen interaction. The human microbiome will be amenable to manipulations to reduce infection risks.

Quote:** “**What is the role of the microbiome and immunologic host factors in the risk of HAI, and how can these be 'manipulated' or included in the prevention of infections?”

On the other hand, medical progress will constantly produce new infectious risks and call for adapted IPC strategies.

## Discussion

Humans tend to rely on established reasoning to solve current challenges. This also applies to the field of IPC. In contrast, systematically exploring a range of possible futures can help manage uncertainties, build resilience, and prepare clinicians to respond to new challenges more rapidly and appropriately. The global IPC experts who participated in this qualitative inquiry saw a positive future with IPC being more integrated into the healthcare systems, reaching beyond the inpatient sector and acute care, making better use of tools for behavioural change and diffusion of knowledge, and benefitting from further scientific discovery while leveraging technology to automate data collection and analysis for both infection surveillance and behaviour monitoring. However, participants also raised concerns about the sustained spread of multidrug-resistant organisms and the slow development of IPC in low- and middle-income countries. In addition, they stressed the need to keep IPC rooted in clinical medicine and present ‘at the sharp end’ where patient care happens.

The participants envisioned the future as predominantly positive. This optimistic attitude is noteworthy. Technological advances are imagined as helping free IPC staff from repetitive office work and allowing them to be more present at the frontline of patient care. Thereby, IPC is projected to ideally evolve in two directions, by increasing the benefit of technology and automation on the one hand and fostering typical human skills such as mindful social behaviour and face-to-face exchange (‘soft skills’). Fears of data breaches, flawed artificial intelligence, and the ongoing difficulties with IT integration did apparently not trigger significant concern. This optimistic view might be due to a natural professional bias among preventionists towards proactive system improvement.

Of note, this survey occurred in the year before the SARS-CoV-2 pandemic. Since then, the views expressed in this survey might have changed substantially. This would justify a repetition of this survey. Interestingly, some participants anticipated the possibility of a large-scale pandemic and questioned whether preparations were sufficient. It is unique to have captured a reflection of expert views of possible futures so close to this global disruption of healthcare that ended up propelling IPC into the mainstream.

IPC “system integration” was a central theme. Participants expressed concern that essential expertise would be lost if core IPC practices were to be overseen by quality managers. This apprehension touches on the contrasting cultural roots of patient safety efforts between quality management and clinically oriented IPC [12]. Participants emphasised the universal importance of clinical experience in IPC and leadership by IPC experts that should not be lost. This view was confirmed shortly after the survey when the pandemic resulted in the sudden urgency to incorporate IPC dimensions into healthcare structures and policies at the institutional, national, and international levels. It should be noted here that the value of IPC expertise goes beyond the healthcare spectrum. As the pandemic continued, the involvement of experienced IPC experts at the government policymaking level was employed in some countries to strengthen the effectiveness of risk communication in the public sphere. Indeed, as Read et al. have highlighted, residents in the UK had a more robust knowledge of IPC after the emergence of COVID-19, suggesting a more general need for IPC education [13].

Despite this unresolved matter, the question about the IPC position in healthcare (Q2: “In whose hands will infection prevention in healthcare settings lay in 2030? Who will be responsible for IPC within healthcare organisations?”) did not produce codes for the leitmotif “Anticipated scientific progress and innovation.” Hence, there might still be an opportunity for organisational research to identify the most effective IPC integration models for different healthcare settings [14].

Various desirable skills among IPC personnel were highlighted, from first-hand clinical experience (nursing, medicine), epidemiology, microbiology, data science, implementation and behaviour science, leadership, project management, organisational management, and complexity science to communication and marketing skills. Considering this wide range of topics, IPC teams—and even healthcare institutions—will be challenged to integrate all this expertise. Astonishingly, the organisational challenge regarding how so many skills would be taught to and mastered by future IPC professionals was not addressed in the ‘open research’ question. In principle, this broad array of know-how and expertise could be covered by interdisciplinary IPC teams with specifically trained and certified experts, as recently showcased [15]; alternatively, healthcare organisations would have to provide access to the corresponding subject experts as transversal resources of the institution. Each solution has advantages and challenges, and these alternative futures have yet to be explored.

The large number of statements matching the leitmotif “behaviour change and implementation” reflects the increasing importance attributed to the behavioural determinants of suboptimal execution of IPC measures. Behavioural aspects were considered key for the sustainability of quality improvement efforts. Techniques mentioned in this survey included role modelling, innovative education methods such as hypermedia^1^ learning and virtual reality [10], nudging [16], implementation science [17], social media [18], monitoring and feedback [19], incentives and reprisal, team science [20], but also classical PDSA cycles.

The potential benefit and the many risks of electronic communication, e.g., the metaverse, were not explicitly mentioned by the respondents [21].

Others have previously addressed the future development of IPC. For example, a recurrent think tank exercise during the biannual Conference on Prevention and Infection Control held in Geneva, Switzerland, has generated three papers [22–24]. The first promoted optimising implementation in IPC [23]; the second focused on technology in the fields of microbiome research, whole genome sequencing, antimicrobial surfaces, and hand hygiene [24]; and the third advocated for global networks to tackle surveillance, antimicrobial stewardship, and research and development [22]. While not strictly using Futures research methods, such think tanks are certainly future-oriented and provide a higher flight-level view of IPC. Some participants of this survey also participated in the Geneva think tanks. Andreas Voss, a vocal advocate for IPC (not participating in this survey), wrote a short opinion monograph on IPC's future, explicitly discussing the post-COVID-19 time [25]. He addressed the shift to long-term care, advocated for harmonised policies across the entire healthcare system, and consequently identified the need for IPC to leave the hospital ‘silo’ behind, extending the scope of IPC to ‘health and lifestyle’ with big players such as Google, Apple, and Amazon and their data analysis capabilities and capacities. The digital and artificial intelligence progress provide new opportunities. The built environment also offers opportunities with its physical (surfaces, controlled airflow) and behavioural (no-touch design) and cognitive properties (‘knowledge in the world’).

This survey specifically addressed the mid-term future of IPC in the next decade. It provides a broad and in-depth view by an international panel of senior IPC experts that was based on a formal qualitative analysis to elicit relevant themes. It forms an exciting starting point and provides a frame of reference on which to build. We hope that the Crystal Ball Initiative will be able to carry this exploration further in an iterative and evolutive manner. For example, as an expression of a more structured and balanced practical approach, the Association of Professional Futurists (https://www.apf.org) has developed a ‘foresight competency model’ that suggests an iterative investigation [26]. Another reality-enhancing method would be to look at the present from the perspective of an already fulfilled future as employed in qualitative research [27]. Furthermore, we could consider including a larger band of experience and specialities beyond IPC, such as human factors engineers [28].

This study has limitations. The response rate was fair, and the number of participants was limited. There was a bias toward participation from the USA, a predominance of experience in acute care, in high-income countries, and an academic physician background. However, the bias favouring innovative thinking and longstanding expertise was deliberate – even if the selection was not exhaustive. An ‘experiential Futures’ research method—accommodating participants in physically simulated future environments [6]—would perhaps have evoked a more thorough and detailed vision of more possible realities for IPC in 2030 than a self-administered online questionnaire [6].

## Conclusions

This first survey within the Crystal Ball Initiative presented a nuanced perspective of potential futures, as elucidated by prominent global leaders in IPC. The outlook was largely positive, harnessing the advancements in technology and medicine to enhance effectiveness and liberating IPC professionals to actively engage at the point of care. This approach, fostered by the active involvement of IPC professionals in both local and global leadership and by adopting a diverse range of skills, will facilitate the seamless integration and expansion of IPC. Future Crystal Ball exercises should aspire to immerse participants more deeply into various scenarios to obtain a more comprehensive picture of the possible realities and involve participants in low- and middle-income countries and professionals who can assess the situation from the outside. Futures research could be pivotal for IPC to plan structures, resources, and education already in the mid-term.

## Data Availability

Data are partially included in this manuscript as quotes from survey responses; the complete survey answers are not made public for anonymity reasons.

## Abbreviations

COVID-19: Coronavirus infectious disease 2019
HAI: Healthcare-associated infections
IPC: Infection prevention and control
IT: Information technology
PDSA: Plan-Do-Study-Act (i.e., cycles of quality improvement)
RFID: Radio frequency identification
Q1 – Q4: Question 1 to question 4
SARS-CoV-2: Severe acute respiratory syndrome coronavirus 2
USA: United States of America
UK: United Kingdom

## Footnotes

1 Hypermedia: Nonlinear medium of information that includes graphics, audio, video, plain text and hyperlinks (Wikipedia).
